# Persistence of a Frameshifting Deletion in SARS-CoV-2 ORF7a for the Duration of a Major Outbreak

**DOI:** 10.3390/v15020522

**Published:** 2023-02-13

**Authors:** Charles S. P. Foster, Rowena A. Bull, Nicodemus Tedla, Fernando Santiago, David Agapiou, Anurag Adhikari, Gregory J. Walker, Lok Bahadur Shrestha, Sebastiaan J. Van Hal, Ki Wook Kim, William D. Rawlinson

**Affiliations:** 1 Serology and Virology Division (SAViD), NSW Health Pathology, Prince of Wales Hospital, Sydney, NSW 2031, Australia; 2 School of Biomedical Sciences, Faculty of Medicine & Health, University of New South Wales, Sydney, NSW 2052, Australia; 3 The Kirby Institute for Infection and Immunity, University of New South Wales, Sydney, NSW 2052, Australia; 4 Department of Infection and Immunology, Kathmandu Research Institute for Biological Sciences, Lalitpur 44700, Province Bagmati, Nepal; 5 Department of Infectious Diseases and Microbiology, NSW Health Pathology, Royal Prince Alfred Hospital, Sydney, NSW 2050, Australia; 6 Central Clinical School, University of Sydney, Sydney, NSW 2006, Australia; 7 School of Women’s and Children’s Health, Faculty of Medicine & Health, University of New South Wales, Sydney, NSW 2052, Australia; 8 School of Biotechnology and Biomolecular Sciences, Faculty of Science, University of New South Wales, Sydney, NSW 2052, Australia

**Keywords:** SARS-CoV-2, ORF7a, indel, outbreak

## Abstract

Australia experienced widespread COVID-19 outbreaks from infection with the SARS-CoV-2 Delta variant between June 2021 and February 2022. A 17-nucleotide frameshift-inducing deletion in ORF7a rapidly became represented at the consensus level (Delta-ORF7a^Δ17del^) in most Australian outbreak cases. Studies from early in the COVID-19 pandemic suggest that frameshift-inducing deletions in ORF7a do not persist for long in the population; therefore, Delta-ORF7a^Δ17del^ genomes should have disappeared early in the Australian outbreak. In this study, we conducted a retrospective analysis of global Delta genomes to characterise the dynamics of Delta-ORF7a^Δ17del^ over time, determined the frequency of all ORF7a deletions worldwide, and compared global trends with those of the Australian Delta outbreak. We downloaded all GISAID clade GK Delta genomes and scanned them for deletions in ORF7a. For each deletion we identified, we characterised its frequency, the number of countries it was found in, and how long it persisted. Of the 4,018,216 Delta genomes identified globally, 134,751 (~3.35%) possessed an ORF7a deletion, and ORF7a^Δ17del^ was the most common. ORF7a^Δ17del^ was the sole deletion in 28,014 genomes, of which 27,912 (~99.6%) originated from the Australian outbreak. During the outbreak, ~87% of genomes were Delta-ORF7a^Δ17del^, and genomes with this deletion were sampled until the outbreak’s end. These data demonstrate that, contrary to suggestions early in the COVID-19 pandemic, genomes with frameshifting deletions in ORF7a can persist over long time periods. We suggest that the proliferation of Delta-ORF7a^Δ17del^ genomes was likely a chance founder effect. Nonetheless, the frequency of ORF7a deletions in SARS-CoV-2 genomes worldwide suggests they might have some benefit for virus transmission.

## 1. Introduction

ORF7a is one of seven non-structural proteins encoded by SARS-CoV-2 which normally localise in the Golgi, endoplasmic reticulum (ER), and cell surface [1]. Of these seven non-structural proteins, three that include ORF7a (ORF3b, ORF6, and ORF7a) are reported to modulate antiviral responses through processes such as the reduction of type 1 interferon [2]. The ORF7a region of SARS-CoV-2 is an ortholog of the corresponding ORF7a region of SARS-CoV, which has previously been documented to be an antagonist of host restriction factor BST2/CD317/Tetherin [3,4]. Host BST2 has been reported to induce and inhibit viral infection by promoting apoptosis of infected cells [5]. During SARS-CoV-2 infections, this apoptotic pathway is inhibited by ORF7a cellular concentrations, resulting in increased SARS-CoV-2 replication and more virus being released [4]. Additionally, the ORF7a accessory protein of SARS-CoV-2 has been linked to the modulation of inflammatory responses via its ability to bind CD14^+^ monocytes and drive a proinflammatory response [6].

Despite the role of ORF7a in immune evasion, deletions at the C-terminus of ORF7a in SARS-CoV-2 have been detected since the early stages of the COVID-19 pandemic. One of the earliest ORF7a deletions, which was reported in March 2020, was an 81-nucleotide in-frame deletion that removed the signal peptide and beta strand sequences of the ORF7a protein [3]. In July 2020, Nemudryi et al. [2] detected a 115-nt disruptive deletion in ORF7a and showed that that this mutation altered host immune responses by enhanced interferon signalling. Placing this sequence in a global context, 189 unique ORF7a variants were detected from the ~181,000 genomes that were uploaded to GISAID at the time. These reported variants were also found to be transient, with few instances of transmission within a host population [2]. This may be due to reduced viral fitness when ORF7a function is altered through in-frame or disruptive mutations, possibly because of a reduced suppression of host innate immunity [2].

**Figure 1:**
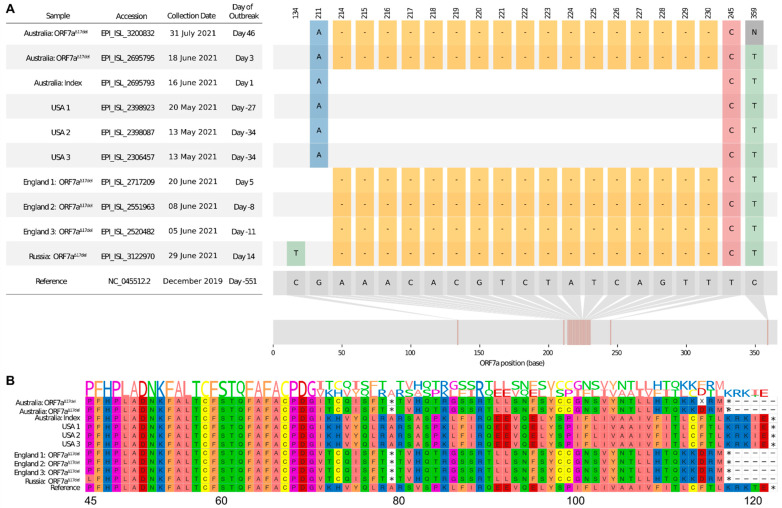
Visualisation of a 17-nucleotide deletion in ORF7a of Delta variant SARS-CoV-2 sequences (ORF7a^Δ17del^) and the corresponding consequences at the peptide level. Sequences chosen for visualisation include an index case of the Australian Delta outbreak (lacking ORF7a^Δ17del^), the first Australian case that possessed ORF7a^Δ17del^ during the outbreak period, an Australian sample from ~1.5 months into the outbreak that possessed ORF7a^Δ17del^, three USA samples (which all lacked ORF7a^Δ17del^) that match the Australian index case, and a selection of four non-Australian samples that possessed ORF7a^Δ17del^, some of which were collected before the Australian outbreak had begun. (**A**) Sample dates of all chosen samples relative to the index case of the Australian Delta outbreak (16 June 2021) and all variants in the sample genomes relative to the SARS-CoV-2 reference genome (created, in part, by using a modified version of snipit, https://github.com/aineniamh/snipit, accessed on 16 August 2021). (**B**) The consequences of ORF7a^Δ17del^ on the amino acid translation of ORF7a, leading to a premature stop codon after amino acid 78 and a truncated peptide sequence (~64% complete). Amino acids 1–44 were identical to the SARS-CoV-2 reference genome in all samples and are not presented. The figure is adapted from [7].

For the first 18 months of the COVID-19 pandemic, there were relatively few SARS-CoV-2 infections in the Australian community compared with global case numbers, particularly in New South Wales (NSW). The first instance of widespread COVID-19 cases in NSW began in June 2021, stemming from community transmission of the SARS-CoV-2 Delta variant (initially B.1.617.2, which was later designated as AY.* Pango sublineages). Outbreak cases soon spread to other Australian states, leading to widespread community transmission. The first virus of this “Delta outbreak” (16 June 2021) exhibited a 100% match, including a full-length intact ORF7a gene (Delta-ORF7a^intact^), when compared with a USA consensus sequence that was the likely progenitor of the Australian outbreak (Figure 1A). However, routine sequencing of subsequent cases identified a 17-nt deletion in the ORF7a gene that spanned genome positions 27607–27623 (“Delta-ORF7a^Δ17del^”, Figure 1). A further analysis of the first outbreak case revealed that a sub-consensus fraction of sequencing reads supported this 17-nt deletion, suggesting a mixed infection in the index case with onward transmission of both viral “subtypes” [7]. Delta outbreak cases soon grew to be characterised by an increased frequency of detection of Delta-ORF7a^Δ17del^, with cases detected until Delta was displaced by Omicron in February 2022, marking the end of the Delta outbreak.

Given that studies from early in the COVID-19 pandemic suggested that frameshifting mutations in ORF7a should be short-lived [2], we undertook this retrospective study to better characterise the spread of Delta-ORF7a^Δ17del^ and explore whether its persistence over many months was unusual compared with other ORF7a variants. We placed Delta-ORF7a^Δ17del^ within the context of all other ORF7a mutations that have occurred globally in Delta SARS-CoV-2 genomes and modelled the effect of the 17-nucleotide deletion on protein structure.

## 2. Materials and Methods

### 2.1. Re-Analysis of Whole-Genome Sequencing Data

Early in the Australian Delta outbreak, we flagged the emergence of Delta-ORF7a^Δ17del^ as a variant worth monitoring and proposed the existence of the ORF7a^Δ17del^ mutation at a sub-consensus level in the index case [7]. This early proposition was based on visual inspection of Oxford Nanopore Technology whole-genome sequencing reads from the first case mapped against the Wuhan Hu-1 reference genome. To quantify this previous result more accurately, we re-analysed the sequencing data from the first outbreak case. Firstly, fast5-format files were re-basecalled with an accurate basecalling model implemented in guppy v6.0.6+8a98bbc (https://community.nanoporetech.com, accessed on 24 May 2022). These data were then analysed using an in-house analysis pipeline (“OAT pipeline”, https://github.com/charlesfoster/ont-analysis-toolkit, accessed on 12 December 2022). Briefly, input reads were filtered by length using nanoq v0.8.6 [8] and mapped against the Wuhan Hu-1 reference genome (MN908947.3) using minimap2 v2.24-r1122 [9]; then, amplicon primers were soft clipped using samtools ampliconclip v1.14 [10]. Variants were called using clair3 [11] with the min_sup_g507 model. Finally, called variants were quality- and depth-filtered, then assembled into a consensus genome using bcftools v1.14 [10]. Using the output of this re-analysis, we determined the estimated variant depth and variant allele frequency of the ORF7a^Δ17del^ mutation in the first outbreak case.

### 2.2. Analysis of Deletions in Global Delta Genomes

Apart from our re-analysis of the first case of the outbreak outlined above, all other analyses in this study were based on downloaded consensus genomes from the GISAID repository. GISAID employs a nomenclature system whereby clades in the global outbreak tree are named based on the presence of key defining mutations. Clade GK predominantly comprises Delta genomes; therefore, we downloaded all clade GK genomes available as a direct download from GISAID (which included 4,223,396 genomes as of 31 May 2022). The full list is available via http://gisaid.org/EPI_SET_220809bs or https://doi.org/10.55876/gis8.220809bs, and the filtered data set is shown in Table S1.

We searched each genome in the dataset for the presence of any deletions within the ORF7a gene using “deletion_detector” (version 0.2.1), which is a custom program that was written in python3 for this study [12]. Briefly, this program aligned each input sequence against the Wuhan Hu-1 reference genome for SARS-CoV-2 using minimap2 v2.24 [9], converted the SAM-format alignment into fasta format using gofasta v1.1.0 [13], and then used custom python code to find the coordinates and lengths of all gaps in a region of the genome of interest. The output file (TSV format) contains the genomic coordinates of deletions within query sequences relative to the reference genome as well as other quality control metrics, depending on the analyses’ input parameters. The date and country of collection for the query sequences were automatically parsed from GISAID headers. All analyses were conducted using parallel processing of input query files. The “deletion_detector” program is freely available as open source software from Zenodo [12] or GitHub (https://github.com/charlesfoster/deletion_detector). The specific command used for this study was “deletion_detector -c ‘27394:27759′ -d ‘27607:27623′ --parse_gisaid --threads 20 input.fasta”.

Clades named according to GISAID nomenclature do not necessarily align completely with other lineage typing systems (e.g., Pango nomenclature, [14]). Clade GK also comprises some other lineages that have independently acquired the defining mutations of the clade. To restrict our survey of ORF7a deletions to only global Delta genomes, we assigned Pango lineages to all downloaded genomes using pangolin v4.06 with pangoLEARN v1.9 [15]. We then imported the results from the “deletion_detector” into R and filtered out all samples that were not assigned to the Delta lineage, were known duplicates, were missing coverage for the entirety of ORF7a, and/or did not have a complete date of collection (YYYY-MM-DD). All code for the generation of summary statistics and the plotting of figures is available as a supplementary document of this manuscript. After this filter, 4,018,216 genomes remained. Summary statistics were then estimated and plotted in R.

## 3. Structural Modelling

Sequence similarities and the secondary structure of an alignment comprising amino acid sequences from the ORF7a region of the Wuhan Hu-1 reference genome and Delta-ORF7a^Δ17del^ were analysed using ESPript3.0 [16]. For the tertiary structural modelling of the ORF7a protein of SARS-CoV-2, SWISS-MODEL software [17] was used with the resolved structure of the ORF7a protein from the SARS-CoV-2 Wuhan-Hu-1 variant (PDB ID: 7CI3) input as a template. The model had a Molprobity score of 1.17, and Ramachandran favored 96.55%. To investigate the potential binding interfaces of SARS-CoV-2 Delta ORF7a with CD14+ monocytes, we performed molecular docking experiments using HDOCK [18]. The structure of the human CD14 antigen was obtained from the RCSB website (PDB ID: 4GLP). The top 10 interactions provided by the software were observed manually, and the model with the highest score and best interaction was selected. The model was further visualised using Pymol, and interactions between the amino-acid residues were calculated.

## 4. Results

### 4.1. Initial Sub-Consensus Frequency of ORF7a^Δ17del^

A re-analysis of the data from the first case of the Delta outbreak recovered a 99.4% complete genome sequence and accurately quantified a sub-consensus frequency of reads that supported the ORF7a^Δ17del^ mutation. Out of a total depth of 167, the ORF7a^Δ17del^ mutation was called with a variant allele frequency of 31%. Given that the sample was originally sequenced on a fresh flow cell, and there were no reads in the concurrent negative control, we are confident that the sub-consensus detection is not the result of contamination.

### 4.2. Frequency of Delta-ORF7a^Δ17del^

Over four million (4,018,216) Delta SARS-CoV-2 consensus genomes downloaded from GISAID met the inclusion criteria for the characterisation of deletions in the ORF7a gene (see Materials and Methods). A total of 4195 unique deletion patterns were detected, and 134,751/4,018,216 (3.35%) genomes in the data set had some form of deletion in ORF7a (Table S1). Approximately 71% of ORF7a deletions were predicted to result in a frameshift and lead to a premature stop codon and truncated amino acid sequence. Most of the deletions were encountered infrequently (median: 2 genomes; IQR: 1–5 genomes), and 19 different deletions occurred in greater than 1000 genome sequences (Table 1 and Table S1). Regarding genomes that had a deletion in ORF7a, generally, only one deletion was detected in a given genome sequence (median: 1 deletion; IQR: 1–1 deletions), but there was a maximum of six deletions found in three genomes. Generally, deletions were detected over short time periods (median: 1 day; IQR = 1–71 days). The longest persisting in-frame deletion was a 12-nt deletion of bases 27617–27628 that was found in 99 genomes, which was first sampled on 11 August 2020 and last sampled on 12 January 2022, a time difference of 519 days (Table S1). Although frameshift-inducing deletions were also generally transient, as expected, some were sampled over a relatively long time period (Table 1 and Table S1). For example, a 64-nucleotide frameshift-inducing deletion that spanned positions 27556–27619 was found in 8945 genome sequences that were sampled between 20 March 2021 and 22 February 2022, a time difference of 339 days. Unsurprisingly, most deletions were detected in only a few countries (median: 1 country; IQR: 1–2 countries) with some evidence of geographical spread when limiting the analysis to deletions occurring in >1000 genomes (median 31; IQR: 19–41 countries).

**Figure 2:**
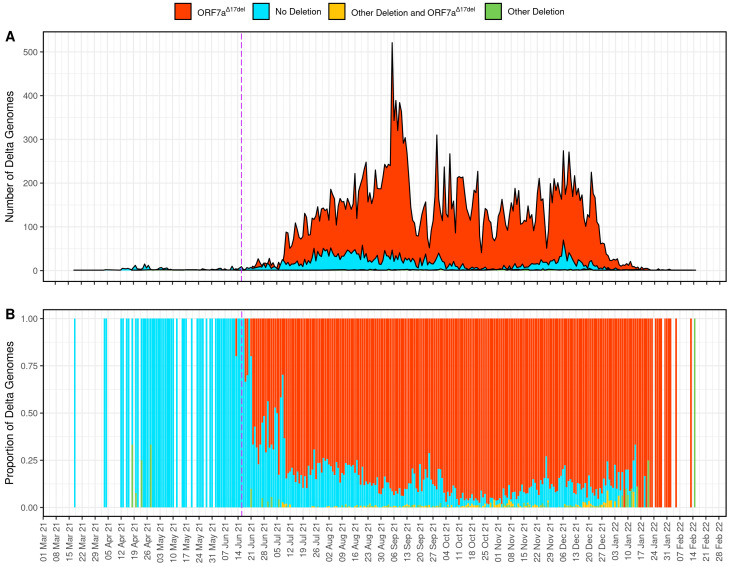
Time series plots of 32,048 genome sequences from the Australian Delta outbreak which show the number (**A**) and proportion (**B**) of genomes per day that possessed the characteristic deletion of 17 nucleotides within ORF7a spanning genomic positions 27607–27623 (ORF7a^Δ17del^), any other deletion in ORF7a, any other deletion in ORF7a plus ORF7a^Δ17del^, or no deletion in ORF7a. The purple dashed line indicates the beginning of the Australian Delta outbreak. Accession numbers for all the samples that were analysed for this panel are available in Table S1 and https://doi.org/10.55876/gis8.220809bs.

The most common ORF7a deletion in the data set corresponded to genomic positions 27,607–27,623, as found in the Delta-ORF7a^Δ17del^ genomes. Of the 134,751 Delta genomes in the filtered data set that have at least one deletion in ORF7a, 28,014 (20.8%) and 166 (0.12%) possessed ORF7a^Δ17del^ as either the sole deletion in ORF7a (Table 2) or as one of several deletions in the gene. The majority of Delta genomes with ORF7a^Δ17del^ in some form (27,912/28,180; 99.05%) originated from Australia (Table 2). Placing these genomes into the context of circulating Delta viruses in Australia between 14 June 2021 (the official start date of the Delta outbreak) and 15 February 2022 (the date of the last detected virus), 87.1% (27,912/32,048) of the Delta genomes that originated from Australia possessed the ORF7a^Δ17del^ deletion (Figure 2 and Table 2).

### 4.3. Structure of ORF7a in Delta-ORF7a^Δ17del^

**Figure 3:**
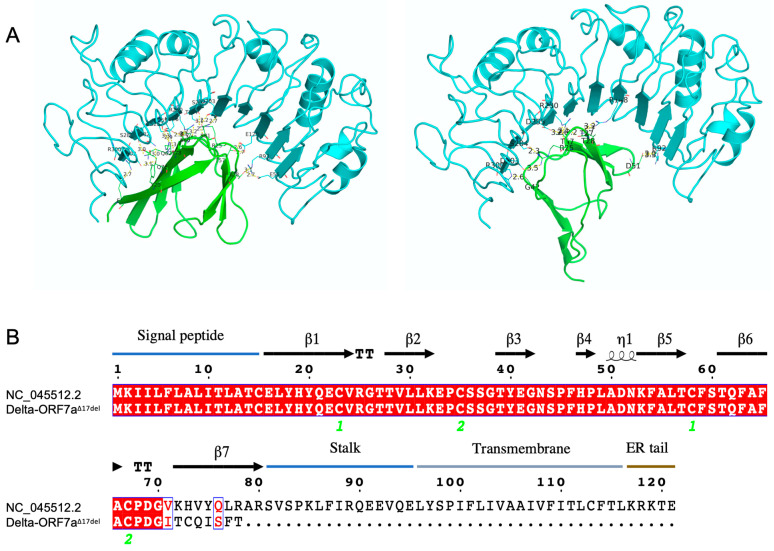
The estimated structure of the ORF7a protein in SARS-CoV-2 and the consequences of a deletion of 17 nucleotides within ORF7a spanning genomic positions 27607–27623 (ORF7a^Δ17del^) on this structure. (**A**) The ORF7a protein from the Wuhan-Hu-1 reference genome (PDB ID: 7CI3, green) docking with human CD14 (PDB ID: 4GLP, turquoise), as modelled using HDOCK [18]. (**B**) The inferred peptide sequence of the ORF7a protein in SARS-CoV-2 which demonstrated the peptide and structural changes within the Delta-ORF7a^Δ17del^ variant. The ORF7a^Δ17del^ deletion occurred after the 6th beta sheet in Delta-ORF7a^Δ17del^, which led to 7 novel amino acids, followed by a stop codon, and caused the loss of the 7th beta sheet, the C-terminal transmembrane domain, and the cytoplasmic di-lysine motif (KRKTE) that determines ER localisation. The alignment was created using Esprit 3.0 [16].

The Delta-ORF7a^Δ17del^ genome encoded normally for the first 70 amino acids of the ORF7a protein. These amino acids included the signal peptide, which was retained in all SARS-CoV-2 ORFs and assisted with protein secretion, followed by 6 beta sheets (Figure 3A). The 17-nt deletion characteristic of Delta-ORF7a^Δ17del^ genomes occurred after the 6th beta sheet, which led to 7 novel amino acids, followed by a stop codon (Figure 1B and Figure 3B), and caused the loss of the 7th beta sheet, the C-terminal transmembrane domain, and the cytoplasmic di-lysine motif (KRKTE) that determines ER localisation. In addition, it also led to the loss of the ORF7a:K119 amino acid, which is polyubiquitinated and is thought to lead to IFN-I inhibition via the blocking of STAT2 [19,20]. The loss of the 50 C-terminal residues is predicted to have a major impact on function. Firstly, the loss of the ORF7a:K119 amino acid is expected to cause the loss of IFN-I suppression in Delta-ORF7a^Δ17del^, resulting in reduced replication titres. Secondly, as ORF7a is normally retained on the cell surface and intracellularly on the Golgi and ER membrane, this deletion, along with the retention of the N-terminal signal peptide, might result in an increased secretion of this protein. Given that the ORF7a ectodomain has been shown to interact with CD14+ monocytes with high efficiency, a systematic increase in the secretion of this protein could result in an altered pathology of this variant [6]. Although protein docking analysis indicated that the ORF7a protein of Delta-ORF7a^Δ17del^ could bind to CD14, the loss of the 7th beta sheet and the K119 significantly altered the proposed interaction sites (Table S2).

## 5. Discussion

Deletions in the ORF7a region of SARS-CoV-2 have been documented since early in the COVID-19 pandemic [2,3,21,22,23,24]. Our analysis of >4 million genomes from clade GK of the SARS-CoV-2 phylogeny demonstrates that ORF7a remains a region where deletions continue to occur, with 3.35% of genomes possessing a deletion in ORF7a (Table S1). Previous studies published during the COVID-19 pandemic documented ORF7a variants, including those that induce frameshifts [2], with evidence of frequent independent emergence. The sustained transmission of these SARS-CoV-2 variants was encountered infrequently and was likely secondary to the reduced viral fitness of these variants [2]. Our results support these findings: 4194 unique ORF7a deletions were detected in our data set of clade GK genomes, but many of these were only sampled once on a single date of collection. Clearly, ORF7a remains a hotspot for deletions in the SARS-CoV-2 genome, and the reason for this phenomenon should continue to be investigated.

Unlike previous studies that were conducted earlier in the COVID-19 pandemic, we show that some ORF7a variants can persist over long time periods, and in some cases, they can persist for more than one year (Table 1 and Table S1). However, these genomes with ORF7a deletions seem to be predominantly sampled at low frequencies relative to the dominant circulating strain. Therefore, the Australian Delta outbreak is unique for several reasons. First, the dominant circulating subtype was an ORF7a variant (Delta-ORF7a^Δ17del^, deletion of genomic positions 27,607–27,623, Figure 2) which accounted for ~87% of Delta genomes in Australia at the time. Second, there was ongoing transmission of this distinctive deletion, with 27,912 genomes sampled over ~7 months (245 days). The true dominance of Delta-ORF7a^Δ17del^ genomes in Australia could also be underestimated in this study since the dataset includes travellers who returned to Australia (who more likely lacked the mutation that was characteristic of the Australian cases) and genomes with potentially incomplete ORF7a sequences (i.e., those potentially lacking coverage at some or all positions 27,607–27,623). Additionally, variant calling software can have difficulties with correctly calling insertion/deletions (indels) [25], and GISAID does not release submissions that contain a frameshift mutation by default (unless it is specifically requested). Finally, the ongoing transmission of Delta-ORF7a^Δ17del^ occurred despite the cocirculation of a virus that would be presumably more “fit” (Delta-ORF7a^intact^) because truncation of the ORF7a C-terminus which leads to a loss of the transmembrane domain has been associated with decreased viral fitness in some (but not all) studies by negating the anti-immune properties of the protein [26].

In concordance with our previous observation [7], the results show that there was a sub-consensus population of the virus in the first case of the Australian outbreak that contained the ORF7a^Δ17del^ deletion. There are two main possibilities that might explain the subsequent persistence and success of Delta-ORF7a^Δ17del^ in Australia. Firstly, the 17-nt deletion could have become (near-)fixed in the outbreak population by chance alone during an initial transmission bottleneck. For example, the sub-consensus population of the virus in the first case(s) with ORF7a^Δ17del^ could have been transmitted to others during infection, became fixed at the consensus level, and then formed part of the ongoing transmission chain. Concurrently, the Delta-ORF7a^intact^ consensus virus might have not been transmitted, leading to local extinction of this viral type. Under this scenario, the net impact would have been ORF7a^Δ17del^‘s persistence in the transmission chain, despite a potential fitness disadvantage, due to a lack of competition from Delta-ORF7a^intact^ viruses and/or other circulating subtypes at that time. Alternatively, the ORF7a^Δ17del^ deletion was either not deleterious to the virus or mildly beneficial relative to Delta-ORF7a^intact^, thereby allowing Delta-ORF7a^Δ17del^ variants to persist and proliferate.

In support of the chance bottleneck explanation, we would expect to see an attenuated growth of Delta-ORF7a^Δ17del^ compared with Delta-ORF7a^intact^ since the expression of functional ORF7a has been reported to inhibit BST2 and lead to an enhanced virion production [27]. However, it is possible that Delta-ORF7a^Δ17del^ retains an ability to bind to BST2 since all but one of the proposed binding sites to BST2 (L17, H19, Q20, D69, and R80) [4] are retained in the truncated protein (Figure 3 and Table S2). Additionally, since the truncated ORF7a protein in Delta-ORF7a^Δ17del^ lacks a transmembrane domain, it is possible that there could be increased secretion of the truncated ORF7a protein relative to Delta-ORF7a^intact^, with unknown fitness consequences. Docking experiments also indicated that the ORF7a truncation abolished binding to the known CD14 binding sites. While other potential binding sites were identified, further experiments need to be performed to confirm these in silico predictions. However, as the Delta-ORF7a^Δ17del^ variant did not appear to associate with increased disease severity in Australia, where it was dominant, it is unlikely that this variant has an increased ability to modulate proinflammatory responses via an interaction with CD14.

Overall, we suggest that no strong fitness advantage from the ORF7a^Δ17del^ mutation is to be expected given the generally deleterious effect of frameshift mutations leading to a partial or full loss of gene function [28]. Despite this, Delta-ORF7a^Δ17del^ viruses were overwhelmingly the infecting virus in COVID-19 within Australia during the outbreak period. The most likely reason for this is an initial founder effect that led to a lack of competition. At the beginning of the Delta outbreak, there was no community transmission of SARS-CoV-2 in NSW and very little transmission in other regions of Australia. Previous instances of community transmission were restricted to relatively small, isolated clusters, and widespread vaccination was yet to be achieved. Consequently, most of the Australian population was immunologically naive to SARS-CoV-2. Additionally, multiple competing variants were not introduced into the community concurrently, partly resulting from strong border controls. These conditions were ideal for the spread of a virus bearing mutations with neutral, mildly beneficial, or mildly detrimental effects that otherwise might have been outcompeted in the presence of other lineages [29]. Indeed, different geographic regions have had isolated outbreaks of SARS-CoV-2 with distinct mutational profiles as a result of founder effects since early in the pandemic [30]. These characteristic mutations are useful for genomic epidemiology by allowing sequences to be assigned to a given cluster or geographic region [31]. It is also important to note that even when mutations do not cause increased infectivity or severity, they can still impact genomic epidemiology by affecting the performance of amplicon schemes of qPCR diagnostics, e.g., [32].

## 6. Conclusions

The rapid proliferation of a particular mutation in the genome of a virus during an outbreak can often trigger concern. Often, this concern turns out to be warranted, such as in cases where mutations clearly led to increased infectivity and/or immune evasion [33,34,35]. However, the phenomena of genetic bottlenecks and founder effects demonstrate that a sudden dominance of a given mutation does not necessarily imply positive selection leading to a more dangerous virus [36]. In this study, we demonstrate that ORF7a deletions have continued to occur relatively frequently throughout the pandemic in viruses from many countries, echoing observations from earlier studies. This trend implies that deletions in ORF7a to SARS-CoV-2 might still have benefits, such as by somehow compensating for more deleterious mutations elsewhere in the genome [37]. Further research into deletions in ORF7a might help understand the ongoing evolution of SARS-CoV-2 and provide useful markers for genomic epidemiology.

## Figures and Tables

**Table 1:** The top 20 deletions in ORF7a of Delta genomes in GISAID clade GK (as of 31 May 2022) ranked by the number of genomes in which they were found. Deletions are summarised in shorthand format in the form of start coordinate, end coordinate, and overall length in the nucleotides. The deletion that characterised the Australian Delta outbreak (Delta-ORF7a^Δ17del^) is represented by “(27607, 27623, 17)” in the first row. Accession numbers for all the samples that were analysed for this table are available in Table S1 and https://doi.org/10.55876/gis8.220809bs.

Deletion	First Date Sampled	Last Date Sampled	Date Span (Days)	Genomes	Countries	Frameshift-Inducing
(27607, 27623, 17)	2 June 2021	13 February 2022	256	28014	27	TRUE
(27556, 27619, 64)	20 March 2021	22 February 2022	339	8945	17	TRUE
(27579, 27581, 3)	22 January 2021	4 February 2022	378	5189	68	FALSE
(27551, 27577, 27)	23 February 2021	15 February 2022	357	3109	30	FALSE
(27692, 27697, 6)	9 March 2021	20 February 2022	348	3108	54	FALSE
(27538, 27628, 91)	4 June 2021	10 February 2022	251	2887	44	TRUE
(27588, 27606, 19)	22 June 2021	17 January 2022	209	2655	8	TRUE
(27555, 27578, 24)	19 May 2021	3 February 2022	260	1916	51	FALSE
(27720, 27721, 2)	12 May 2021	22 February 2022	286	1859	46	TRUE
(27553, 27621, 69)	9 April 2021	4 February 2022	301	1636	33	FALSE
(27554, 27621, 68)	12 April 2021	4 January 2022	267	1585	21	TRUE
(27694, 27700, 7)	9 February 2021	12 January 2022	337	1538	38	TRUE
(27548, 27623, 76)	21 June 2021	10 January 2022	203	1418	13	TRUE
(27548, 27554, 7)	11 January 2021	17 January 2022	371	1290	31	TRUE
(27578, 27623, 46)	4 April 2021	31 January 2022	302	1135	34	TRUE
(27555, 27624, 70)	5 April 2021	2 February 2022	303	1115	28	TRUE
(27564, 27586, 23)	9 June 2021	12 January 2022	217	1044	17	TRUE
(27553, 27553, 1)	22 April 2021	18 January 2022	271	1007	35	TRUE
(27555, 27633, 79)	26 July 2021	31 December 2021	158	1003	7	TRUE
(27695, 27700, 6)	16 April 2021	25 January 2022	284	992	35	FALSE

**Table 2:** All countries that generated genomes possessing the deletion that characterised the Australian Delta outbreak (ORF7a^Δ17del^) during the outbreak time period, as determined by analysis of all Delta genomes in GISAID clade GK (as of 31 May 2022). The genomes either possessed ORF7a^Δ17del^ as the sole deletion in ORF7a, or it was one of two or more deletions in the gene. The table is ranked by the number of genomes with ORF7a^Δ17del^. The percentage of Delta genomes submitted to GISAID during the time period that had ORF7a^Δ17del^ is given to two decimal places. Note: a date span of 0 days suggests that genome(s) were collected on a single date. Accession numbers for all samples analysed for this panel are in Table S1 and https://doi.org/10.55876/gis8.220809bs.

Country	Genomes with ORF7a^Δ17del^ Deletion	Total Genomes in Period	Percentage of Genomes with Deletion	First Date Sampled	Last Date Sampled	Date Span (Days)
Australia	27912	32,048	87.09	13 June 2021	13 February 2022	245
USA	102	1,402,035	0.01	14 July 2021	20 December 2021	159
England	76	865,173	0.01	2 June 2021	23 December 2021	204
Brazil	24	40,686	0.06	20 September 2021	17 January 2022	119
France	17	114,469	0.01	20 October 2021	27 December 2021	68
India	7	73,032	0.01	18 September 2021	3 January 2022	107
Slovenia	6	27,823	0.02	19 October 2021	10 December 2021	52
Thailand	6	7775	0.08	11 November 2021	19 December 2021	38
Netherlands	4	44,346	0.01	16 November 2021	4 January 2022	49
Scotland	4	103,198	0.00	28 October 2021	17 December 2021	50
Colombia	2	4525	0.04	22 November 2021	19 December 2021	27
Japan	2	95,139	0.00	30 August 2021	28 September 2021	29
Mexico	2	23,422	0.01	12 December 2021	12 December 2021	0
Romania	2	5884	0.03	30 September 2021	30 September 2021	0
Russia	2	7647	0.03	29 June 2021	2 November 2021	126
Bahrain	1	1893	0.05	11 November 2021	11 November 2021	0
Canada	1	97,883	0.00	20 October 2021	20 October 2021	0
Chile	1	8486	0.01	28 November 2021	28 November 2021	0
Croatia	1	14,217	0.01	4 September 2021	4 September 2021	0
Israel	1	18,019	0.01	6 November 2021	6 November 2021	0
Latvia	1	5220	0.02	13 October 2021	13 October 2021	0
Lithuania	1	15,449	0.01	30 October 2021	30 October 2021	0
Luxembourg	1	9493	0.01	29 November 2021	29 November 2021	0
Malta	1	19	5.26	8 January 2022	8 January 2022	0
Peru	1	6575	0.02	28 October 2021	28 October 2021	0
Vietnam	1	2423	0.04	16 November 2021	16 November 2021	0
Wales	1	98,861	0.00	24 August 2021	24 August 2021	0

## Data Availability

All genomes analysed for the purpose of this study are available via http://gisaid.org/EPI_SET_220809bs and https://doi.org/10.55876/gis8.220809bs. The ‘deletion_detector’ program written for the purposes of this study is freely available as open-source software from Zenodo (https://doi.org/10.5281/zenodo.7049120) or GitHub (https://github.com/charlesfoster/deletion_detector). Code for the generation of summary statistics and the plotting of figures is available as a supplementary document of this manuscript.

## Supplementary Materials

The following supporting information can be downloaded at: https://www.mdpi.com/article/10.3390/v15020522/s1, Table S1: All inferred deletions in the ORF7a region and associated quality control metrics of 4,018,216 Delta SARS-CoV-2 clade GK genomes, downloaded from GISAID on 2022-05-31; Table S2: Inferred binding sites between SARS-CoV-2 ORF7a and human CD14. The inferences were made using HDOCK based on the ORF7a sequence from the Wuhan-Hu-1 reference genome and the Delta-ORF7a^Δ17del^ variant. “analyse_output_data_supplementary.R”: code written in R to generate the summary statistics and plot the figures used in this manuscript.
