# Supplementary material for: Persistence of a Frameshifting Deletion in SARS-CoV-2 ORF7a for the Duration of a Major Outbreak
Source: Viruses. 2023 Feb 13;15(2):522. doi: 10.3390/v15020522 (PMC9966144; doi:10.3390/v15020522)
Supplement: Supplementary file 1 [file viruses-15-00522-s001.zip › viruses-2202222-supplementary.pdf]

Table S1. All inferred deletions in the ORF7a region, and associated quality control metrics, of 4,018,216 Delta SARS-CoV-2 clade GK genomes downloaded from GISAID on 2022-05-31.

Note: Table is too large for this document and can be accessed from:

- doi: 10.6084/m9.figshare.21012535

Table S2. Inferred binding sites between SARS-CoV-2 ORF7a and human CD14. The inferences were made using HDOCK based on the ORF7a sequence from the Wuhan-Hu-1 reference genome and from the Delta-ORF7a$^{\Delta 17del}$ variant.

**Genome**	**ORF7a Site**	**Distance (Å)**	**CD14**
NC_045512.2 (Wuhan-Hu-1)	E16	2.7	R300
NC_045512.2 (Wuhan-Hu-1)	Y20	3	N282
NC_045512.2 (Wuhan-Hu-1)	Y20	3.1	D303
NC_045512.2 (Wuhan-Hu-1)	S81	2.2	D203
NC_045512.2 (Wuhan-Hu-1)	R25	2.7	D203
NC_045512.2 (Wuhan-Hu-1)	T57	2.6	E120
NC_045512.2 (Wuhan-Hu-1)	T57	2.7	E120
NC_045512.2 (Wuhan-Hu-1)	T28	3.1	R93
NC_045512.2 (Wuhan-Hu-1)	T28	2.7	E56
NC_045512.2 (Wuhan-Hu-1)	Q76	3	S280
NC_045512.2 (Wuhan-Hu-1)	Q62	3.3	N226
NC_045512.2 (Wuhan-Hu-1)	R78	3.3	S257
NC_045512.2 (Wuhan-Hu-1)	N43	2.7	T197
NC_045512.2 (Wuhan-Hu-1)	S60	3.1	D255
Delta-ORF7a$^{\Delta 17del}$	G42	2.6	R300
Delta-ORF7a$^{\Delta 17del}$	R25	3.5	D303
Delta-ORF7a$^{\Delta 17del}$	R25	2.3	S284
Delta-ORF7a$^{\Delta 17del}$	T57	2.9	D255
Delta-ORF7a$^{\Delta 17del}$	D51	3	R92
Delta-ORF7a$^{\Delta 17del}$	T28	2.7	R148
Delta-ORF7a$^{\Delta 17del}$	T28	3.2	R148
